# Supplementary material for: Look at me! An exploratory study of supported eating interactions in long-term neurological care
Source: Int J Qual Stud Health Well-being. 2025 May 26;20(1):2508948. doi: 10.1080/17482631.2025.2508948 (PMC12107641; doi:10.1080/17482631.2025.2508948)
Supplement: LatchemHastings_Look_at_me_accepted_manuscript.docx [file ZQHW_A_2508948_SM5556.docx]

**COVER PAGE**

**SUBMISSION FOR:**

International Journal of Qualitative Studies on Health and Well-being

**MANUSCRIPT TITLE:**

Look at me! An exploratory study of supported eating interactions in long-term neurological care

**AUTHOR:**

Dr Julie Latchem-Hastings

School of Healthcare Sciences, Cardiff University, UK

[LatchemJM@cardiff.ac.uk](mailto:LatchemJM@cardiff.ac.uk)

**Look at me! An exploratory study of supported eating interactions in long-term neurological care**

**Abstract:**

**Introduction:** Care homes are synonymous with aged care; however, many younger people also reside in care homes, often because they have complex needs caused by neurological conditions. Of this population, some people require support to eat. People in care homes consider mealtimes as central to their care experience but repeatedly report dissatisfaction with them. This paper examines what makes for positive or negative supported eating interactions (SEI) between care staff and people with neurological conditions aged 18-65.

**Methods:** The paper draws upon semi-structured interviews conducted with residents and healthcare staff exploring the role of food in the care of adults with neurological conditions in long-term care settings.

**Results:** Six core themes (1. Time and timing, 2. Individualized support and care(ing), 3. Choice and autonomy; 4. Core clinical knowledge and skills; 5. De-humanizing Practices; and 6. Environment) drawn through reflexive thematic analysis were identified.

**Conclusions:** There was significant parity between resident and staff considerations regarding the essence of what makes up a positive or negative SEI. Most core principles for delivering positive SEI’s fall within the knowledge and skills of individual healthcare staff. However, the findings on time and environment require organizational support to enable staff to deliver the best SEI.

…..

**Key words**

Neurological conditions, long-term care, mealtimes, supported feeding, eating, dining.

**Introduction**

The prevalence of major disabling neurological disorders, such as Dementia, Parkinson’s Disease and Acquired Brain Injury, has been increasing globally over the past 30 years (Feigin et al. 2020). An ever-increasing number of people have complex needs, with neurological conditions being the leading cause of disability-adjusted life-years (Global Burden of Diseases Study 2016; Neurology Collaborators, 2019). The complex needs of people with a neurological condition cannot always be supported at home, and long-term care in specialist care homes can provide an alternative place of care for this population (Latchem-Hastings 2023).

Maximizing the quality of life of people living in long-term care is a critical issue for residents, their families, and care providers. Good nutrition/hydration and enjoyable mealtimes are critical for the health and well-being of everyone (Department of Health 2007) and are particularly important for people who reside in care homes, for whom mealtimes can be the highlight of the day, providing a social focal point (Royal Society for Public Health 2009; Barnes et al. 2013; Philpin et al. 2013; Watkins et al. 2017; Namasivayam-MacDonald et al. 2017; Morrison-Koechl et al. 2021).

**Mealtime experience**

The literature base examining the ‘mealtime experience’ in long-term care settings is extensive - but largely concludes that what people in long-term care in the western world want regarding their ‘mealtime experience’ appears to be ‘universal’ with residents requesting *“hot home cooked meals served in the dining room at the table with everyday plates in a quiet atmosphere seated with friends and neighbors”* (Adams et al. 2013, p. 3). Despite the surface simplicity of this request, dissatisfaction with mealtimes is common, with research reporting concerns about mealtime experiences from residents themselves, families, and caregivers alike.

Concerns about food quality, nutrition, hydration, and mealtime experiences for residents in health and social care facilities have persisted in the UK and internationally for over 30 years (Manthorpe and Watson 2002; Merrell et al. 2012; Philpin et al.2014; Josefsson et al. 2018; Davies et al. 2022), with research, user-led initiatives, and formal reviews repeatedly providing devastating summaries of mealtimes. In many care facilities mealtimes are task driven, seen and conducted as a ‘feeding activity,’ failing to enable choice or create a positive experience for residents (Reimer and Keller 2009; Barnes et al. 2013; Older People’s Commissioner for Wales, 2014; Watkins et al. 2017). Overwhelming dining room environments, fixed menus, rushed meals and poor staff support, including poor support for those unable to feed themselves are amongst the many issues identified internationally as contributors to poor mealtime experience in long-term care (Carrier et al. 2009; Chadbury et al. 2013, Lowndes et al. 2015, Watkins et al. 2017, Nielsen et al. 2024). This research, however, predominantly focused on long-term care settings caring for people aged 65 years and above.

**Supported eating**

Neurological conditions inflict a range of physical, sensory, and cognitive impairments that can dramatically impact how people with neurological conditions eat, in terms of the mechanism of chewing and swallowing (Burgos et al. 2018; Panebianco et al. 2020; Halfpenny et al. 2021) and feeding themselves, the physical mechanics of using cutlery or hands to pick up food, bring it to the mouth, and take food from a utensil (Clarke et al. 2018; Wäckerlin et al. 2020). Therefore, many require support from another person to eat. Such support may be focused on one component of eating a meal – for example, cutting up food into small pieces for a younger adult who has suffered a stroke, and is left hemiplegic, unable to use one arm. Others with more profound impairments (e.g. through spinal cord injuries) or for those who suffer from neurological fatigue as part of their condition (e.g., as seen in Multiple Sclerosis) can require much more extensive support to eat, reliant on being fed – having food and drink brought up to their mouth by others (Martinsen, Harder and Biering-Sorensen 2008).

Feeding oneself is a central personal task that denotes adult independence. This sense of adult independence can be lost by reliance on others for the placement of food into mouths or a sense of infantilization experienced by those able to eat independently but unable to do so tidily, tipping food onto oneself, or the area around. Therefore, mealtimes are unsurprisingly raised in care guidelines as an opportunity to respect residents’ dignity or to undermine it (Older Peopale’s Commissioner for Wales, 2014).

For those who require support to eat, the relationship between the person providing food (feeding) and the person eating food is intimate (Martinsen, Harder and Biering-Sorensen 2008). Therefore, working with people with complex neurological conditions requires awareness of the social and emotional aspects of eating, the management of eating-related risks, and interpersonal and technical skills (Wilmot, Legg and Barratt 2002; Heaven et al. 2013).

Many adults in nursing homes and other types of long-term care settings require support to eat; however, despite this common care activity, supported eating has received relatively little research attention. Snippets of the expansive literature base examining the ‘mealtime experience’ as a whole, highlighted above often identified the importance of ‘assistance with eating at mealtimes’ from both a resident (Harstäde et al. 2025) and staff perspective (Nielsen, Harstäde and Sandgren 2025), but does not examine what these needs are or unpick what might make up positive or negative support. In contrast to the research attention paid to the mealtime overall, there is only a small body of literature spanning 40 years, which specifically examines supported eating in long-term care. This literature includes examination of the impact of carer turnover (the number of different staff who support residents to eat) on consumption (Backstrom et al. 1987), assessment of the time taken for staff to assist ‘physically dependent’ residents to eat accompanied by the measurement of oral intake (Simmons and Schnelle 2006) and health outcome comparisons of ‘assisted hand feeding’ versus nasal gastric tube feeding (Chou et al. 2020). This research predominantly focusses on people aged 65 and above with dementia and is largely quantitative - assessing food consumption rather than the quality of the experience for the person being cared for.

Research examining the lived experience of people being supported to eat in long-term care settings is helpfully synthesised in a meta-ethnography (Martinsen et al. 2007) which examined 10 papers published between 1989 – 2005. This meta-analysis reports two key themes which characterize supported eating interactions – ‘feeding as task’ and ‘feeding as relationship.’ Of the included papers, all, bar one, focused on the supported eating of older adults (aged 65+), most with dementia, and drew predominantly on staff experience, not that of residents. The papers examine ‘feeding problems’ of care home residents (Athlin, Norberg, and Jansson, 1989; Kumlien, and Axelsson 2002, Michaelsson, Norbery and Norberg 1987 and Pasman et al. 2003), nursing and care staff perceptions of resident behaviors around mealtimes (Athlin, Norberg, and Asplund 1990, Pearson, Fitzgerald and Nay 2003), supported feeding organization and feeding practices (Kayser-Jones and Scheel 1997, Kumlien and Axelsson 2002, Pelletier 2005 and Pierson 1999) and the nature of interactions during supported feeding (van Ort and Phillips 1992). Despite the paper topics, none focus entirely on the supported eating interaction (SEI), but akin to the mealtime experience literature, look at mealtime more broadly.

One study, Martinsen, Harder and Biering-Sorensen (2008) examines the meaning of ‘assisted feeding’ for adults with spinal cord injuries. The focus of their study is on the construction of meaning and the relational dynamics between the person being supported to eat and the care professional rather than examining the SEI itself.

What makes for a positive SEI and exactly what it contains is, therefore, largely undefined and underexplored, and the experiences of younger adults (aged 18-65) in long-term care on this subject are unreported. Therefore, this study explicitly explores what makes positive and negative SEI’s for younger adults (aged 18-65) residing in neurological long-term care settings.

**Methods**

**Data collection**

This paper draws upon a food-focused study ‘FEAST’ – Food, eating and drinking in long-term care: sharing practice to transform care conducted between 2020-2023 by the author. This study examined the role of food in the care and rehabilitation of younger people residing in specialized long-term care neurological settings. Originally devised as an ethnographic study, the study required redesign to enable it to be conducted during the COVID-19 pandemic when in-person access to care facilities for research was prohibited. Collecting data predominantly online via a conferencing platform, semi-structured interviews were held with 17 staff members working in eight different long-term neurological care settings in England and Wales. Once physical access to care facilities became possible, interviews with a further 20 staff members and eight residents were conducted face-to-face in one neurological long-term care setting in England.

Data were collected from a range of health and care staff working at eight different neurological long-term care settings and residents who had experienced supported eating interactions at different care institutions through their care and rehabilitative journeys. Tables 1 and 2 provide demographic summaries of the participants.

**[INSERT TABLE 1 HERE]**

**[INSERT TABLE 2 HERE]**

**Data analysis**

Interviews were transcribed verbatim and de-identified. Each participant was given a pseudonym.

Braun and Clarke’s reflexive thematic analysis (RTA) (2022) was used to analyze the datasets. Thematic analysis is a *“method for developing, analysing and interpreting patterns across a qualitative dataset, which involves a systematic process of data coding to develop themes”* (Braun and Clarke 2022, p.4), in which the development of themes is the ultimate analytical purpose. RTA is particularly useful for ‘Big Q’ qualitative studies like the FEAST study which are ‘fully qualitative’ in nature – using qualitative tools and techniques within a qualitative paradigm (Kidder and Fine, 1987).

Braun and Clarke (2022) expressed the process of RTA through six phases: dataset familiarization, data coding, initial theme generation, theme development and review, theme refining, defining and naming, and writing up. These phases are not intended as rigid linear rules to follow but as clear guidelines. ‘Writing up’ as a phase, highlights this – with the work of refining themes continuous to the last. In practice, toing and froing between phases to hone themes was extensively required during the analysis of the data presented here.

In addition to the dataset above, a member checking process was conducted in which the initial themes identified through analysis were shared and discussed with three residents (two females and one male) residing in one neurological long-term care setting. Themes were discussed and residents who were asked to express whether they felt the themes a) matched their experiences and b) were complete. The discussion of the themes prompted the sharing of additional memories, thoughts or experiences. These discussions have been added as additional data.

**Ethics**

Ethical approval for the FEAST study was obtained by Cardiff University’s School of Medicine Ethics Committee on 31^st^ March 2020. Informed consent for participation in the study was obtained. All participants who participated in the study had the capacity to consent on their own behalf. For interviews conducted online consent was recorded verbally and via the completion of an online consent form. For interviews conducted face to face, participants completed and signed hard copy consent forms.

**Findings**

In interviews across data sets, perspectives on what makes for ‘good’ and ‘bad’ SEIs were offered in tandem, with the opposite of the ‘good’ examples being bad and the opposite of the bad being good, rather than defined reporting of what was good followed by what was bad. Interwoven into responses were challenges to achieving the good and core frustrations about repeated poor practices. Mitigations of the challenges were sometimes, although not always, offered by the participants.

Six central themes are depicted in Figure 1. which denote the good and bad of SEIs with adults with neurological conditions in long-term care settings. These are 1. Time and timing, 2. Individualised support and care(ing), 3. Choice and autonomy, 4. Core clinical/care knowledge and skills, 5. De-humanising Practices and 6. Environment.

**[INSERT FIGURE 1 HERE]**

1. **Time and Timing**

Data were entrenched with temporality and a plethora of temporal concepts were used repeatedly and consistently to describe the positive and negative aspects of SEI’s.

**1.1 Time**

Taking time or conversely, a lack of time was continuously identified as central to driving high- or poor-quality SEI. Below Victoria, a dietitian, and Marie, a care assistant, indicate the impact of staff being time-pressured.

*There just isn’t enough time for these people, the people who are assisting patients or care staff. They end up rushing or they don’t have, not that they don’t have the patience, but they’ve got five million other things to be doing at that time. In order to give that person the most safe and enjoyable experience, they’re limited by time. –* ***Victoria, dietitian***

*I know on days you’re very rushed, I guess the conversation probably doesn’t flow as quick and people are looking for the next resident that needs feeding and they’re trying to get things done as quickly as possible. That’s not their fault, they’re just a little bit stretched for time. -* ***Marie, Care assistant***

How this can play out in practice at mealtimes is further exemplified by Lara, a speech and language therapist who described a mealtime she had recently observed where a staff nurse was trying to support one resident to eat, monitor two others eating, and was being interrupted by other staff members throughout:

*[Resident] was being fed by a senior member of the team. Over the other side of the table there was somebody who needed some supervision, but just more of an eye kept on him, and then there was another gentleman who was feeding himself, but probably also needed a bit of support, so she was trying to do all of that. At the same time, she was answering questions from a healthcare assistant who had come up asking about ordering incontinence pads, and there was somebody from maintenance who was also asking her a question. Now, it’s rubbish for her because she was trying to do a job, it was really* *rubbish for the residents because that’s not a pleasant mealtime in my book. It’s, it’s a conveyor belt, it’s getting food in you, it’s not, that social quiet time, let’s sit and enjoy and talk about food, and you know smell it, just you know chill out a bit, just enjoy it.* **Lara – Speech and Language therapist**

**1.2 Timing (Pacing and pausing)**

Healthcare staff interviewed drew links between ‘timing’, ‘pacing’ and ‘pausing’ and the quality and safety of a SEI.

Speech and language therapist Angela, talking about her observations of staff supporting residents to eat, highlights how waiting, pacing, the order of food, and intermittent introduction of fluid are all important aspects of the SEI. She explains:

*They [healthcare assistants] concentrated a lot on the diet and then I'm thinking are you offering fluids? They would offer the drink and it would be an afterthought. So, it’d be he's got some residue in his mouth there, you could wait for him to swallow it or maybe you could try and use the fluid, that seemed to be something that I needed to remind them about. You know, where’s his drink, so just timings –* ***Angela, Speech and Language Therapist***

Alongside staff observation, residents shared their experiences. One resident, reflecting on a time in her rehabilitative journey when she needed support to eat, said:

*When I was being fed, right at the beginning [following a stroke] you didn’t want someone to rush you. Being fed, it’s a completely alien concept as an adult. It was almost stressful, each mealtime was stressful. The food arrives and where is the person going to feed me? Food can go really cold really quickly –* **Carmen, Resident.**

Here, Carmen overtly states not wanting to be rushed when she is being supported to eat and also highlights the importance of timing in relation to food and care support arriving together so that food can be provided hot.

Staff describing positive SEIs almost always included some sense of pacing in their descriptions. Tara, a Therapy assistant said simply *“let them just take their time, and don’t rush, I think that’s the most important thing”,* while Occupational Therapist, Madeline talking about the complexity of delivering a positive supported eating experience. In doing so, she draws out a series of nuanced temporal considerations within the interaction.

*You have to be mindful of so many things. […] How much have you put on the fork or spoon, and have you given them enough time. Would they normally stop for a few moments and have you given them enough time. Would they normally stop for a few moments and have a drink, or am I just on this train of, we’ll keep going until the plate is empty. Would they naturally have paused, even if they’re not going to be making conversation, would there be built in rest periods?* – **Madeline, Occupational Therapist**

On asking interviewees what key skills they thought care staff needed to deliver a positive SEI Therapy Assistant, Ruth immediately stated:

*The carers need to understand that you’ve got to go at the patient’s pace, rather than the carer’s pace. –* **Ruth, Therapy Assistant**

Mixed with getting the timing right for a person and taking time with them, this act of appropriate time-giving was also considered by staff to communicate to the person they were supporting that they were valued. A chef, Bobbie and Jenny, an Occupational Therapist, expressed this as follows:

*[not rushing] so that person is valued and it's not just a task to tick box through.* – **Jenny, Occupational Therapist**

*It’s just about being patient, you’re not going to get it all in them and then run back and then that’s it, you’re done. Mealtime is protective, it’s precious and you sit there. Like if you went out for lunch, you wouldn’t whoof it all down you and then that would be it. So, yes, it’s just about being patient and persisting with them and just making them feel comfortable.* – **Bobbie, Chef**

**1.3 Presence** **(being present)**

Being in the moment, not distracted, and focused on the person being supported to eat was also repeatedly highlighted as a positive element of supported eating interactions. Gill, a nurse, makes this point.

*It’s about being in the moment with the person who you’re supporting […] It’s not about talking over people because staff can get quite a banter going at mealtimes. Some of that is entertaining for residents, but it tends to [take the] focus off residents, when actually it should be a more personal time for them. So, I think it’s crucial that staff try to get into the moment with that person.* ***– Gill, Nurse***

The importance of presence was reiterated by a nurse manager, Hannah – *“Basic table manners, from them [staff], so, if you are sat with someone, you are solely engaging on them, you’re not on a handset catching up on your notes.”* Occupational Therapist Jenny said, *“It’s having that full focus on that individual, making sure communication is happening between the person who's being fed and the person feeding.”*

Lara, a Speech and Language Therapist, provided a detailed description of a positive SEI where taking time, presence, and significant interaction were central:

*I was up on [ward] watching one of the healthcare assistants talking through a meal with somebody who was showing her absolutely no idea that he was listening to her but she was saying it anyway. So she was saying “this is what you’ve got”, she said what was on the plate, she didn’t go “I don’t know what it is, what’s on here, anyone know what’s on here, what’s puree today” none on that, and she did it so normally. She brought it up to her, she smelled it, she gave it to the resident to smell, he did smell it. She’s giving him some spoonfuls and she’s saying “ok you’ve now got some potato, I hope it’s alright, I don’t think it’s got any lumps in it”, and she’s just giving this little commentary. Sometimes she goes a little quiet and she was just kind of looking around and looking at him and then she’s going “I hope you’re ready for something else” and it was so nice, it was quite moving to watch because it was like she was feeding a member of her own family. And you’re thinking, you can do that, watch, watch what she’s doing, it’s just brilliant.* **Lara – Speech and Language therapist**

**1.4 Resident-initiated**

In addition to ensuring that SEIs are given time, are paced, include appropriate pauses, and rest, staff discussed the importance of residents being able to initiate eating – to start and stop as they wanted and needed. Selina, a healthcare assistant, explained how she always encourages residents to indicate when they want more food.

*I always say “you let me know when you’re ready for some more and we can get that sorted rather than just me deciding you look ready” –* ***Selina, Healthcare Assistant***

Selena also explained how resident-led/initiated pacing of a meal can be achieved even if they are unable to communicate verbally.

*Let them lead. Even if they can’t verbally say yes, I’d like some more please or I’m ready.  They just open their mouth –* ***Selina, Healthcare Assistant***

In contrast, not being resident-led was linked to the delivery of a very poor, even dangerous, supported feeding practice. Angelica, a resident who intermittently requires support to eat herself and frequently observed another resident unable to communicate verbally, explained:

*The amount of times I have seen people shovel food in before he’s even had time to swallow or breathe. They don’t offer him a drink between mouthfuls. I look at these people and think is this is how you eat? You just shovel it. It is a lot nicer when people are patient and give people a break between mouthfuls. At least when I’ve had someone assist me they can say “are you ready for another one”, he needs somebody who can pick up his facial cues. Has he finished chewing? Sometimes he stores the food in his cheek pockets. If people aren’t looking out for that it can be really dangerous and a horrible experience. –* ***Angelica***

Temporality–Time and timing play a central role in the delivery of a very good or equally bad SEI. Pacing, taking time, and being present are all key to the provision of a positive SEI; conversely, rushing, not giving residents breaks and rests in-between mouthfuls, and being distracted and not focused on the person being supported to eat contributes to a very bad SEI.

1. **Individualised support and care(ing) for the person**

Along with time and timing, residents and staff identified that for SEIs to be positive, support had to be personalized and individualized.

**2.1 Knowing the person**

Interviewed residents were quick to highlight the importance of being known by the people supporting them to eat. This ‘knowing’ has multiple components. Mark, a resident, explained the importance of care staff understanding and knowing their physical capabilities and, crucially, how those had changed over time.

*Knowing the persons’ body – before I didn’t have a strong swallow, and now it is much better – I guess that’s about knowing the patient* – ***Mark, resident***

The importance of ‘knowing’ a resident and their needs in relation to mealtimes was operationalized in many settings using mealtime-based communication aids for staff called ‘meal mats. ’ A ‘meal mat’ is a personalized table mat which has specific information about the needs, likes and dislikes of a resident on it, to help care staff see at a glance, how they can best support them at mealtimes. Occupational Therapist Madeline explained what this method of ‘knowing’ offered:

*So the OT section which would say, this person uses this type of cutlery, or keep the plate warm and the speech and language therapist would put what diet type and fluid type they should have. All that information being there, it was easy for staff to check, it was very particular to that person, and it could have their preferences and their favourite meals, all these bits of information, that was helpful.* ***Madeline –*** ***Occupational Therapist***

Healthcare Assistant Caroline highlighted that meal mats provided further information beyond cutlery requirements and food and drink texture.

*[The meal mats could include] how they eat, the risk of them choking, aspirating etc. The basics to tell them “Make sure you eat slowly” or if the patient is able to do it themselves, “encourage the patient as much as possibly, to be independent.”* ***Caroline, Health care Assistant***

The importance of individualization in supported eating was readily recognized by staff who identified that this was best achieved by ‘knowing’ residents but also identified that ‘knowing’ required staff to support a resident regularly.

*It’s just that very individualised approach, just knowing the patient I think is so important. That can be an issue when we have bank staff who just rotate around or come once a month and do the feeding. I think it is especially important to really know the patient, know their pace, how they respond, how much time to give them after – and you just learn that by doing that every day and getting to know the patient.* ***Martina, Therapy Assistant***

Along with the physical and clinical knowledge and understanding of staff, resident Carmen expressed the importance of being seen as a person and not a clinical task. She said:

*She really made it look like it wasn’t a job. She made it look like she was facilitating my needs. She didn’t make me feel like I was a baby whilst spooning food into my mouth. She recognised me not the job. Yes her job was to feed me but it’s a bigger task. Recognising the person in that task. –* ***Carmen, resident***

**2.2 Enabling independence**

Linked to Mark’s earlier comment regarding care staff knowing what his physical capabilities were, residents and staff also highlighted that a significant part of what makes for a good, SEI includes staff enabling residents to do as much as they can for themselves.

For example, Angelica, a resident noted:

*Being encouraged to do things where they can, I think that’s really important. Some people, simply because it takes too long, will not allow Darren [resident] the option of feeding himself. He just needs a bit of assistance at his elbow. But just because they think they don’t have the time they don’t encourage him to eat on his own. One lad up here is now eating on his own where people have encouraged and enabled him to do it. It’s such a big thing. –* ***Angelica, Resident***

And nurse manager Liz said:

*Liz: It’s promoting the abilities that still remain. If that person has still got use in their right hand, don’t just assume or take over and stop that kind of progress or ability or independence from going anywhere. It’s supporting that continued ability. -* ***Liz, Nurse Managers***

Staff, particularly therapy staff also noted this aspect repeatedly in their descriptions of ‘good’ SEIs. For example Occupational Therapist Clara explains:

*It’s giving the person an element of choice about what they have next to eat, so encouraging them to be involved with the activity, even if they’re not physically able to lift a spoon. And from there, if you need to feed them, feed them, if they can do it themselves, give them the opportunity to do as much as they can before they fatigue. That would be the main things –* ***Clara, Occupational Therapist***

**2.3 Making the mealtime meaningful**

Individualized support, however, was not considered purely in functional or therapeutic terms. Speech and Language Therapists repeatedly spoke about the use of ‘tasters,’ the giving of small amounts of food to residents “*where consuming larger amounts of food is tricky and then taste and food is used more as a pleasurable component than a life sustaining component.”* **(Mary, Speech and Language Therapist).**

The positive impact of supporting residents unable to eat for fuel and nutritional purposes with the provision of tasters was highlighted repeatedly. Speech and Language Therapists enthused about a device, a ‘Bio zoom’ which turns small amounts of food into a froth that retains a strong flavor of the original substance but as a foam, which enables it to be safely consumed for those unable to swallow, even the smallest mouthful of solid food.

Speech and Language Therapist Mary provided an example where the use of the Bio zoom provided enjoyment to a resident and his mother, who was able to support her son.

*One of our chaps, his mum was devastated that he'd stopped [eating] because he always showed so much enjoyment with food. And, when we got it started, we got her to come in and she did it [using the Bio Zoom with him]. He does really show enjoyment opening his mouth widely for more. So, not only is he enjoying it, but she's enjoying doing something with him that he enjoys. I always love that about ‘taste for pleasure’, the social aspect of feeding is huge.* ***Mary, Speech and Language Therapist***

Here, a ‘mealtime’ has been recreated for the resident through individualized clinical support of a speech and language therapist and made meaningful through the involvement of family members.

Exploring further the importance of mealtimes as meaningful and recognizing individual contexts surrounding food and the SEI Speech and Language therapist Lara reflected on how she talks to staff in training sessions about the importance of recognizing individuals’ relationships with food, how their neurological deficit might impact their enjoyment of mealtimes, and the role staff have in trying to enhance the experience.

*It’s not just about saying to care staff, when you feed somebody, this is how you do it, it’s about saying, people think of food in different ways. And when you’ve got somebody who’s not interacting in a normal way, and looking and speaking and listening to you, maybe you can get an ‘in’ with showing them some food, getting them to smell it and just talking about it to try and get that memory.* ***Lara, Speech and language therapist***

Alongside Lara, Rosy, a psychologist, discussed the opportunity mealtimes offered for meaningful interaction and stimulation for people whose cognition is severely impacted by their neurological condition. Rosy describes how an SEI can be personalized to bring meaning to a resident’s day.

*That’s one thing I do observe, when you’ve got patients who are bed-bound whose eyes are closed most of the time, a carer will still come in and say, “I’ve got your dinner”, they describe what’s on the plate, they’ll pick up a spoon full of it and say, “I’ve got some mash here”, so they’re talking them through it. Even then, they might not be aware of that, but they’re talking them through it and they’re respecting them and they’re making that time of their day significant, ‘cause most of the time they’ll just lay in bed asleep potentially. So, I think, for me, mealtimes are the most interactive things that some people can have.* ***Rosy -*** ***Psychologist***

Carmen, a resident, added to this, reflecting on the importance to her interacting while eating saying:

*When I was being fed, there was this student nurse and I got on with her so well and she made the effort to make it more individual. When food gets put down and then they bugger off, that’s disconcerting and doesn’t help your mood. It’s nice to talk just in general [when eating a meal].* ***Carmen, resident.***

Staff interviewed also recognised the importance of the social interaction they could provide to residents through the SEI, and this too needed to be provided on a very individual basis. Selina, a healthcare assistant, explained,

*How much I talk to a resident, I think it depends on the resident because some residents need to completely concentrate on what they’re doing. Some are quite happy to have a chat, so I always just let the resident decide. I may make a conversation and then if they respond well to it then that’ll sort of set the tone. If not, then I think right, they need to concentrate, so it’s very individual.* ***Selina, Health Care Assistant***

Individualized support, clinically and socially, and residents feeling seen as a person and not a task within the SEI, is an essential part of providing a positive experience.

1. **Choice**

Ensuring and enabling choice(s) within the SEI were also identified as central to good practices by most staff, and all residents interviewed.

**3.1 Choice within the supported eating interaction**

Both staff and residents described how choice could and should be enabled during the SEI itself. Occupational Therapist Clara comments on the relationship between choice and overall mealtime satisfaction saying:

*It would be nice if patients had more choice in what on their plate they ate next, rather than that food chosen by the person feeding them. Then I think that would probably influence enjoyment of the meals. –* ***Clara, Occupational Therapist***

Marta discussed how choices could be made regarding the environment surrounding the meal, as well as within the SEI itself.

*I’m quite passionate about there being choices, in terms of whether it’s where you want to sit, what would you like to drink, and giving options around that. But also, in the actual interaction of feeding, do you want another spoon, offering rather than just feeding. The bugbear for me is the automatic spoon to your face.* ***– Marta, Psychologist***

**3.2 Consent (and choice)**

Marta, reflecting on the importance of offering choice in SEI’s, discussed how the constant provision of choice goes hand in hand with consent, asking for consent before delivering any medical, care, or therapeutic action.

*Being from a position of always being a psychologist and never a HCA or in a support worker role, I always felt that because you always have to have a boundary, there’s an element of always asking for consent when supporting someone to eat. There always needs to be “can I give you another spoon? do you want a spoon of porridge or do you want a spoon of yogurt, or would you like a bite of toast?”. I appreciate this takes way longer than most people would have to support somebody’s meal, but I wouldn’t feel comfortable just feeding an adult in that way. –* ***Marta, Psychologist***

**3.3 Choice restriction**

Above, and across earlier themes presented, staff recognize that the provision of ‘choice’ in a SEI may take longer than mechanically feeding someone, and residents being supported to make choices and given choices through the supported eating interaction is influenced by time. For staff who consider their job is to ‘feed’ and not to support residents in eating and enjoying their mealtime, choice(s) can become limited to residents.

The impact of neurological conditions on eating, complicate mealtimes further. Here Marta highlights how the cognitive challenges people with neurological conditions can have mixed with the time pressures of staff can collide with ill effect for those who need support to eat.

*If my job is to get this meal delivered, choice gets removed. With people who perhaps cognitively might need some support, they struggle with initiation, so, although they can get started on a meal, they might struggle to keep going, so then it’s important to continue to give choices there, rather than assuming that the person has finished with their meal, and take the meal away, when actually they just needed to be prompted and asked, “are you finished, would you like some more?” So really, just thinking about choice-making, and the individual’s needs. Like we were saying earlier, it’s very individualised; eating and drinking after a brain injury does have very different issues. –* ***Marta, Psychologist***

Reflecting further on the impact of time on choice, Marta explains that the reasons underpinning this issue go beyond immediate staffing availability and time pressures and have a broader political and onward service provision underpinning. She explains an interconnection between choice within supported eating interactions, time, and onward care:

*Time, also, is an issue there. Like I said before, if I was feeding somebody and I was offering them all those choices all the time, it would take me in excess of 45 minutes to feed. And we all know that. In clinical settings, we have to document when we’re discharging someone, how long a meal takes, and that will influence the care they’re given. So, Social Services are not going to fund, they’re not going to be able to support anybody who takes longer than 45 minutes for a meal, because that’s longer than a shift is expected to take during a care call. So, then, rather than us being able to support this person’s meals, we’re having to think about, can we make them eat quicker, or do they need more supplements and less meal? And that’s really sad. That is due to funding and staffing. Or that is why people don’t offer the choices and the spooning is happening, because that’s what makes it quicker. The same as somebody who can feed themselves, but it might take them longer to use their own spoon, might be fed instead, because it will be quicker.* ***Marta, Psychologist***

The importance of choice in the provision of good SEI was discussed at length by participants, with clear examples of how this can be achieved in practice. The provision of choice however was highlighted as constantly under threat by time pressures and could be removed altogether to enable discharge home where care needs needed to be packaged so they could be achieved within the length of a short care ‘call.’

1. **Core clinical and care skills**

The importance of knowing the person, their physical abilities, and clinical needs was explored, in part, in the theme ‘individualized support. ’ However, the complexity and multiplicity of the needs of residents with neurological conditions and the knowledge required by staff need to be examined in the context of SEIs. Both staff and residents discussed the core clinical and basic care skills and knowledge needed to support residents with neurological conditions to eat.

**4.1 Safety as fundamental to the experience**

The risk of choking for residents for whom their neurological condition has impacted their swallowing was often at the forefront of the minds of the participants. Staff and residents alike highlighted how minimizing the risk of choking and maximizing safe and effective swallowing were critical in the delivery of a positive supported eating interaction. Conversely, unsafe-supported eating practices were at the top of the list in descriptions of negative SEI.

Residents spoke of their own experiences of choking and the need for staff to be clinically competent when supporting residents in eating. Carmen reflected:

*I’ve been through all the stages of diet, I was on pureed food [following a stroke]. Because of my muscle weakness even though I’m on normal diet now I have issues with moving food around my mouth. I’ve chocked several times and I’ve had to have people put their fingers in my mouth and stuff and it is quite scary. Having someone who understands the mechanics is vital actually –* ***Carmen, resident***

Safety considerations for residents during SEIs extend beyond the risks posed by swallowing difficulty. For residents with cognitive and behavioral challenges, their ability to control impulses around food or even be able to identify what is and is not food could be impacted by their neurological condition.

*[Some residents can have] very poor insight, a lot of impulsivity, so when that burger comes in, if it hasn’t already been cut up it might go all the way in! Eating non-edible objects, keeping those out of their sphere [is needed]. We've also had people not recognising food as food, and the challenge of getting [food] near them -* ***Mary, Speech and Language Therapist***

For staff working with residents whose neurological conditions had caused behavioral changes, ‘safety’ during SEIs included considering safety of staff and other residents, as cutlery and cups could easily become missiles. ‘Risk management’ included the considerations expressed by Mary, a speech and language Therapist:

*So, spoons, forks, cups, the weight of the cup, where do they throw them? Staff having to get in close proximity of them to help with feeding. Food and drinks being thrown, what temperature are they? How big, how hard? So, there's lots of risk management.* ***Mary, Speech and Language Therapist***

**4.2 Managing risk – key knowledge and skills**

All types of staff included safe-supported eating practices within their consideration of good and bad eating interactions. However, the most extensive discussions about safety came, unsurprisingly, from speech and language therapists, whose job roles and expertise explicitly assess and rehabilitate swallowing.

Residents and staff alike highlighted that there were a series of basic but critical skills that staff needed to understand to support residents to eat as safely as possible. These included basic upright positioning:

*Darren needs to be sat fully upright otherwise he’s a choking risk –* ***Angelica, Resident***

*The real fundamentals of sitting someone upright, making sure they stay upright, making sure they’re alert, all those sorts of things* – **Ffion, Speech and Language Therapist**

Many staff also highlighted the importance of ‘assessment’, continually assessing and (re)evaluating the needs and abilities of residents with regards to their ability to chew and swallow. Nurse Manager Hannah reflected this saying:

*Does someone like to eat quite fast? Okay, what are the risk behind that? Is there anything we need to do to manage this? So, someone might be high risk of choking, staff knowing exactly what signs to look out for, not just [actual] choking. –* ***Hannah, Nurse Manager***

For some residents, risk elimination was not an aim. Instead, risks were acknowledged by residents and health care professionals together, and a series of practices were designed and put into place to minimize the risks associated with eating orally. Speech and Language Therapist Ffion, describes her ‘ideal’ SEI delivered by a carer who was skilled enough to minimize risk but provide suitable interaction during the meal also. She explained:

*My ideal supportive feeding is purely that person doing everything they can to minimise the risk. I’m probably skewed in the sense that they’re the things that I focus on. However, I think the really good ones are the people and the carer who I have seen that can do all of that, but still make that a nice interaction, or a human interaction with that person. I find it really awkward sometimes because I would always say [to care staff] “don’t ask that person questions when they’ve got food in their mouth, don’t talk to them whilst they’re eating because that’s going to cause them to become distracted or talk and lose control of that bolus”, but it’s the ones that can find that really nice balance. It’s building the rapport and having those sorts of conversations and getting little bits of conversation in-between mouthfuls but then also being aware of, right, that person’s chewing at the moment, they’re trying to trigger a swallow, this is not the time.* ***Ffion – Speech and Language Therapist***

Speech and language therapists and, on occasion, other health and care staff interviewed expressed frustration regarding how speech and language therapy recommendations for achieving safe supported eating interactions could be misunderstood by other staff and seen as limiting the eating experience for residents. This could happen particularly when a resident was only just managing to eat or drink orally and the margin between safe and unsafe oral intake was small.

*I was very aware that I am pushing the boundaries here with somebody that isn’t eligible for oral intake on paper, shouldn’t be able to manage this but actually in reality, he is, so let’s go with that, and let’s limit the risks in other areas. He will probably need to be in his room, not distracted, somebody with him, etc. etc. We looked at positive risk management and him being able to have something that wasn’t pureed once a week and see how he goes with that. All positive, but then they [care staff] always went up that next level, “so why can't he eat in the dining room with everybody else, why can't he eat outside his room”, because he silently aspirates as well, that was the issue, so they couldn't see [distractions made his swallow unsafe].* ***Angela, Speech and Language Therapist***

Speech and language therapists also stressed the frustration that nursing and health care assistants could be more cautious in SEIs with residents who showed outward signs of challenges with swallowing (for example, coughing and dribbling) but were less cautious with those who ‘silently aspirated’ – people for whom food is going into the trachea rather than the stomach. They explained that modifying diets should be the last resort, with all other strategies explored first – including having mealtimes away from others if needed. Angela explained:

*I'm a big believer in making the modification of the diet the last strategy we use to ensure a safe swallow, and people are sometimes not aware of the other strategies i.e. around supportive feeding, posture, environment, everything else, that can lead towards a safer swallow because I feel like that's the least restrictive.* ***Angela, Speech and Language Therapist***

**4.3 The problem of medicalisation - clashing clinical and social needs at mealtimes**

The clinical need of residents during mealtimes is unescapable, but meeting these clinical needs risks medicalizing primarily social activity. Staff, particularly therapists, recognized these and suggested practices to mitigate this. Lara, a speech and language therapist, reflected the following:

*If somebody is feeding someone else, if they want to have a drink with them, then I think at the very least have a drink. I can understand somebody not necessarily wanting to eat their lunch when they’re care feeding you know, people aren’t always the tidiest of eaters, and it can be a little bit off putting, watching somebody eat with it all kind of around their faces and coming back out again and all that kind of stuff, but at least if you have a drink there, it’s a little bit more like, it’s a little bit just normal*

*Researcher: So that sharing of food*

*Yeah, ‘cause we are medicalising. That’s what I hate about the bloody hair nets. We’re making something that is an everyday pleasure into something that is again, this is something I’m doing to you because I have to, and you have to eat because you have to get nutrition. Not this is your meal, enjoy it.*

Within the description of the happening remembered earlier by Lara in Theme 2, there is a mixing of the clinical (and at times the ‘dirty’) cutting across what is a predominantly social aspect of the day. This was demonstrated by the healthcare assistant asking the nurse about incontinence pads while the nurse supported residents to eat within a dining space. The presence and use of clinical items in a dining space, at mealtime in the scene, the administration of medications during mealtimes with medicine trolleys, and nurses wearing medication tabards entering the dining space have also been observed in ethnographic work in long-term neurological care settings (Latchem 2017). Both the presence of medicalized items and the practices that went with them, along with medical-based discussions, serve to medicalize mealtimes.

Both staff and residents alike recognized that safety and the management of risk were central to a good SEI. The inescapable dangers of eating for some residents predominantly require certain clinical and care practices to be put in place; however, additional medical practices could also coincide with mealtimes. In doing so, the social nature of eating and the opportunity mealtimes present for socializing can be usurped.

1. **De-humanising practices**

Practices that de-humanized residents or could be viewed or experienced as undignified, peppered interviews with staff and residents. Residents recounted aspects of the interactions they had experienced themselves or witnessed happening to others, highlighting a series of problematic practices.

**5.1 Infantilisation**

Angelica, a resident, described witnessing infantilising practices during supported eating interactions:

*I’ve seen so many people talk to Darren – he’s a grown man, he’s in his 50’s – “come on now Darren open up”. I said to this care assistant – “excuse me, you do realise he’s a grown man, talking to him like that dehumanises him”, she looked at me like I was from outer space. It can be a very hard thing to be a grown adult and have someone feed you but if it’s done in the right way it can make you feel cared for. Someone is taking the time to make sure they are feeding you properly, they aren’t spilling it all over you, they’re wiping your mouth if you’ve got a bit of food there. It’s almost, it could be considered quite an intimate thing, someone feeding you.* ***Angelica, Resident***

Such practices were also mirrored by descriptions within staff interviews:

*I've seen people go into adults like a child “well here it comes”, and you just think, oh dear.  Why are you doing that?* ***Selina, Healthcare Assistant***

Alongside infantilising actions and language, the use of certain artifacts for adults with disabilities at mealtimes could be seen as infantilising in and of themselves.

*They [healthcare assistant] took the spouted beaker, put it in her mouth, and she [healthcare assistant] was talking to someone else, and this poor woman was obviously drowning. Spouted beakers have always been the bane of my life, I hate them. But for some people they work, I'll qualify it, I understand, but I avoid them. I don't think there's anyone, if you served them a drink in that, would be like “oh lovely”.* ***Mary, Speech and Language Therapist***

In addition to beakers, residents with neurological conditions can sometimes require modified cutlery and modified food, including pureed food, to enable them to eat independently or to swallow their food safely. Owing to dexterity and coordination challenges, ‘clothes protectors’ (a form of plastic bib) are often placed over them. The assemblage of these challenges and the artifacts used to ‘help,’ all add to the potential for residents to feel infantilised.

‘Mess’ made during eating, because of its relation to being child-like could also be seen as undignified by staff. Consequently, they highlight the importance of reducing the indignity of messy eating. Selina, a Healthcare Assistant, reflected the following:

*Putting too much food on the spoon, leaving food around peoples’ mouths, not covering their clothes if it looks like they're potentially gonna make a mess with their food.  Just undignified.* – **Selina, Healthcare Assistant**

Conversely, one resident highlighted that what is and is not dignified is individualized. For example, she expressed her need for choice about whether or not she received ‘help’ when eating.

*I really hate it when people just touch me. Just because you think I need help, if I chose to have food down me that’s my choice, stop touching me. That makes me really mad.* ***Carmen, Resident***

**5.2 Position, speed and attention**

The position staff were in when supporting residents to eat, and the speed at which they brought food to residents’ mouths was repeatedly highlighted by residents and staff as de-humanizing, inappropriate practices. Residents and staff spoke about staff standing over a resident and ‘shovelling’ food in, leading to offering spoonfuls of food too quickly, without giving residents a break in-between.

My pet peeve you know, standing over them, feeding, let’s just shovel it in, shovel it in, ‘come on, have another mouthful’, and then shout to the sister that so and so hasn’t had their meds and then shovel it in, you know, I think it’s dehumanising. But when they actually pull up a seat and they feel like they’re at the table, I’ve seen that work quite positively – ***Madeline, Occupational Therapist***

*You’ve got others that will stand up and feed and you can see it’s about just getting it done quick. You’ve got people that have got a passion and then you’ve got people that are there just doing a job. –* ***Ruth, Therapy Assistant***

*“Shovelling happens all the time and it makes me really angry. This person has not asked to be in this situation. Treat them with some dignity. .”-* ***Carmen, Resident***

Here, the important temporal aspects of delivering a positive SEI highlighted in theme 1, time, and timing, are not fulfilled and de-humanize as a result. Angelica, a resident, reflects further on the impact of ‘shovelling’ and what the act of ’shovelling communicates to residents.

*I think the biggest thing for the carer to remember is that the person they are helping is a human – and how would they like to be treated if they were needed help. Don’t just treat them like an inconvenience and shovel food in. –* ***Angelica, Resident***

Residents and staff also noted stark examples of a lack of attention being paid to the needs of a person, exemplified by one resident reflecting on an incident when he was fed via a percutaneous endoscopic gastrostomy.

*“I remember being offered food being a PEG feed and staff leaving it [a tray of food] on my table. It was torture” –* ***Mark, Resident***

Unable to speak without the aid of communication technology, this resident was unable to tell staff he could not eat orally at the time, and food was left in front of him.

Residents recognized that experiences of poor SEIs could negatively impact how they felt about mealtimes, and both residents and staff alike talked about how resident consumption of food could be heavily impacted by who was supporting them to eat. Angelica reflected:

*I think the biggest thing is to not make a big thing about it. Yes, you are feeding that person and you might need, carry on having a conversation, carry on watching the tv show together, try and make it as normal as possible. The person who is having to be assisted, they could come to dread food and mealtimes. I’ve seen Darren refuse to eat because it’s been the wrong person. –* ***Angelica, Resident***

In contrast to the dehumanizing practices described above, descriptions of good, supported eating practices were provided as a remedy. Bobbie, a chef talked passionately about how staff approached residents and how they positioned themselves physically while supporting a resident to eat, had a direct impact on maintaining the dignity of a person or degrading it, and in turn, impacted whether residents ate their meals.

*I think it’s just being on their level. I think when someone is needing assistance when they eat, it’s kind of a bit embarrassing, it’s, your dignity. It’s gone a little bit and I just think they need someone, to sit by the side of them, not in their face and just be calm, chilled. I don’t think people really understand what it’s like to have to go through that if you haven’t, we haven’t been there, but I think it is a hard one to get right. When it is [right], you see the results in their eating. A lot of people […] are in your face, and almost like a bit scary, with this massive fork coming at you, and you’re like, oh my God, what is this? Then they’re put off by their food. So, I think sat there, and talking to them, just talking to them and making them feel so comfortable, that they’re like, “yes, I am going to eat this, this is nice now.”* **Bobby - Chef**

Therapy assistant Ruth commented further on the positive role of getting positioning and levels of interaction right have on creating a mealtime experience.

*Ninety percent of them (health care assistants) are doing it really well, they’ll sit at the patients’ level, they’ll have a normal conversation with them, while they’re having a meal. So, it’ll be like a proper meal setting, “Oh, what did you do in group today?”* **Ruth - Therapy Assistant**

Practices making up SEIs can lead to de-humanizing those being assisted and risk dignity. Remedies to this, however, are described simply, with positioning considerations, being present, taking time, and interacting gently being central to maintaining dignity and delivering a positive supported eating experience.

1. **Environment**

This paper focuses on the SEI itself, but as exemplified across themes, the interaction does not sit in a vacuum and is impacted by many things outside. The immediate environment surrounding the person being supported to eat was highlighted as a crucial element in the delivery of good or bad interaction.

**6.1 Dining area stimulation and distraction(s)**

Residents and staff noted the impact of ‘stimulus,’ particularly sounds around the person being supported to eat. Angelica, a resident, considered the importance of sound and noise levels in this context:

*Stimulation – if there is too much noise it would make it a lot harder to concentrate. I think that is what it comes down to – eating requires concentration. When we eat – we just chatter away but when you’re recovering from illnesses, it’s different. It’s not overly noisy in our dining room so we do have the radio on for some background noise so it’s not silent. Eating in silence would make you feel like you’re at school when you’d be shouted at for making too much noise. I think it’s really important that there is some noise but it’s not overly stimulating or distracting.* **Angelica, Resident**

Madeline, an Occupational Therapist, further explained that some people who need support to eat can be easily distracted and need verbal and physical prompts to support them to eat. Environments that limit distractions are therefore important for enabling positive, safe, and effective SEIs.

*For some people, you would like an environment that’s free from distractions. There can be the need for a lot of prompting, or just sometimes that hand over hand support [to lift fork or spoon to the mouth] or, just touch their hand, so they remember to lift it again. If other people are walking by, it's so easy for people that struggle to eat, to get distracted. If they've got distracted, they can be slower, and you do see it, you do still see people kind of coming along and going “oh you know, why aren't you hungry today”, and taking the plate away. They probably would have finished it if you hadn’t kept distracting them all the time.* ***– Madeline, Occupational Therapist.***

Above, Madeline also links the environment (in this case, a quiet space where others are not coming and going through or past) and the amount of food residents consume, highlighting that some residents are able to eat more when not distracted. However, Angelica, a resident noted that eating together at mealtimes was an important social event (and therefore inherently noisier) but could also result in residents eating well:

*We notice here that if we do a social activity that involves eating, people tend to eat more. People who wouldn’t normally eat with other people do come and join. To keep people apart makes the whole things a chore. Eating should be enjoyable –* ***Angelica, Resident***

Speaking about a fellow resident who would be considered as someone who could become distracted, Angelica also highlighted how eating with others in fact helped him concentrate on his eating, saying:

*When Darren is brought in, because he sees us eating, it almost focusses him to eat. If he’s sat on his own, or next to the TV, he gets distracted by that. Definitely being sat with other people eating is definitely better for him. –* ***Angelica, Resident***

Speech and Language Therapist Erica explained that for some people with neurological conditions, they will not eat at all unless they are eating with others.

*I’ve been to some places where the residents get upset if someone’s not eating, because, cognitively, they’re not necessarily orientated to the understanding of the situation and they refuse to eat, unless [the other person is also eating] –* ***Erica, Speech and Language Therapist***

For residents whose consumption was negatively impacted by busy environments, removing environmental distractions could often be ‘solved’ by providing residents with meals in their rooms or taking them into a dining space where/when no other residents were eating. As highlighted in theme 4, core clinical and care skills prioritizing safe oral consumption over social interaction with others is central to a good SEI as defined by both staff and residents. However, eating in isolation could also be viewed as negative.

Recognizing the need to balance the clinical, social, speech, and language therapists Mary, agreeing with the above offered some practical ways of reducing distractions within the social space of the dining room to reduce the need for residents to eat alone. She suggested:

*For those that do have attentional difficulties, moving them to a side or facing where maybe they have one person in front of them, but not a whole room, to avoid that distractibility. -* ***Mary, Speech and Language Therapist***

Mark gave a very different perspective on the impact of eating with others on his consumption of food. He explained:

*I can go into the dining room but I choose not to. I know the residents and staff really well and they try, and do, make me laugh. I have an uncontrollable laugh and it makes me choke! When I eat with my friends, that’s the worst! –* ***Mark, Resident***

Ironically, as a consequence of his sense of humour and rapport with others, Mark ate alone in his room.

**6.2 The mealtime set-up**

The area immediately around or in front of the person was also considered by staff and residents to be an important part of setting up the supported eating interaction, as Therapists Clara and Judy and Therapy Assistant Tara explained:

Tara begins her description by focusing on the comfort of the resident ahead of their mealtime, saying:

*They’ve been to the toilet, they’re settled, they’ve got a comfortable chair. […] then I would position them so I would face them and I would make sure that I’ve got a table in front of me, and I would discuss what food was on the plate. –* ***Tara, Therapy Assistant***

Clara an Occupational therapist continues:

*So first thing would be set up the environment and the person [ask them] can they see the plate in front of them, can they see the cutlery, introduce them to the task you're doing, so they know what to expect, and then think about where you position yourself, so you're not towering over somebody, or if they've got a visual field deficit, you're not coming in from a blind side and there's a magic spoon appearing in the air. Talk about those things. –* ***Clara, Occupational Therapist***

Speech and Language Therapist, Judy adds further considerations regarding positioning:

*A lot of our residents have quite large chairs. Some with tray tables. So, thinking about where they've positioned the food, so that they can see it. Where the person feeding, or facilitating feeding is at the same height, and that any conversation is directed at them, or they're part of that experience. –* ***Judy, Speech and Language Therapist***

And resident Angelica, reflecting particularly on the needs of another resident added:

*The set-up Darren responds so much better to is when someone puts a small table in front of him and puts the food in front of him and encourages him to eat [independently]. If he can’t, if they sit on the side that they find it easier to turn his head they can talk to him and help him. I think that makes a big difference. –* ***Angelica, Resident***

As can be seen in the quotes above, the physical position of the assisting staff member, the position of the resident within their environment, and the positioning of food and utensils in relation to the resident are all considered an important part of the quality of the interaction. Positioning of the resident being supported to eat and the staff member who is assisting has also played a role in other themes reported here – important in terms of safe positions to enable safe and effective swallow (theme 4) and making up part of practices that humanize or dehumanize (theme 5).

This theme outlines how the immediate environment surrounding SEIs impacts the quality of the interaction and residents’ consumption of food. On the one hand, eating with others is an important social activity that can enhance mealtimes’ experiences and support residents in eating well. Conversely, in the context of neurological conditions, eating with others can be problematic, becoming a distraction, and in turn, reducing overall consumption. The individualization and manipulation of eating environments for residents with neurological conditions makes a significant contribution to ensuring positive SEIs.

**Discussion**

The aim of this study was to provide detailed description of what makes for a good and bad SEI for adults with neurological conditions aged 18-65 in long-term care settings. This is the first study to date to 1. focus entirely on the supported eating interaction with this population, 2. in this setting and 3. bringing together the experiences of both receiving and giving food in a SEI, from the resident and the health and care professional perspectives.

The main thematic ‘headlines’ explain the essence of what makes up a good interaction as temporal – taking time and being present, providing care and support which is individualized, ensuring choice, managing risk, the immediate environment surrounding the interaction and ensuring care practices maintain dignity and do not de-humanize.

Although the mealtime experience in long-term care is the subject of a large body of research, focus on the SEI itself has continued to be illusive. The main contributors in this area are to be found in Martinsen et al. (2007) meta-ethnography examined in the introduction. In addition, the only other paper found examining the experience of younger adults with neurological conditions is a further study by Martinsen et al. (2008), a phenological study with adults with spinal cord injuries. Aspects of the findings presented from this study do mirror findings from these previous international studies conducted in Europe, the USA and Australia, over the past 20-30 years, primarily with older people residing in aged care settings. These include 1. the challenge being short of time poses to quality SEIs (Kayser-Jones and Schell 1997; Pearson, Fitzgerald and Nay 2003), 2. the importance of maintaining independence within the SEI (Kayser and Schell 1999), 3. the need for core practical skills, such as the ability to read interpret non-verbal cues (Pierson 1999), 4. the recognition and persistence of de-humanizing practices (Pearson, Fitzgerald, and Nay 2003) and 5. the importance of managing the mealtime environment by reducing multiple stimuli (Athlin, Norberg, and Jansson 1989 and Kayser and Schell 1999).

Uniquely, this study reported a series of new findings as well as those which add further nuance and detail on this topic. The importance of resident choice within the interaction featured consistently through the dataset of this study and formed an entire theme, yet the aged-care literature base is largely silent on this component. The tensions between the medical and the social, which also features so strongly in the findings presented here, especially in theme ‘Core clinical and care skills’, were also not drawn out by the aged care studies.

Many of the international studies discussed above reported observations of poor core practices, including unsafe feeding practices and practices that continually threaten dignity. Healthcare professional participants in this study reported time in their careers when they had seen the continuation of these bad practices, and residents reported recent experiences of being on the receiving end of such practices. Healthcare professional participants were able to clearly identify what good and bad practice was. They displayed knowledge and skills, concern, and passion for the provision of good care that went well beyond that reported in any of the past international studies highlighted above.

In addition, healthcare professional participant viewpoints differed in this study in comparison to some of that reported in the aged care literature. Nursing staff taking part in Pearson, Fitzgerald and Nay’s (2003) study thought that maintaining resident independence within the SEI was important for resident ‘morale’ rather than as part of rehabilitation or maintaining one’s dignity so readily understood 20 year later by participants in this study.

**Future research**

Whilst acknowledging that there is much that is shared and applicable across long-term care settings and populations highlighted in the discussion of similarities to and gaps in the international literature above, the details and additional nuances presented in this study indicate the importance of separately researching what happens in long-term care settings with different resident populations. The repeated findings across place and time discussed, while evidencing rigor of the research presented here and that of the past, also raises challenging questions about why poor practices known about for so long persist.

This study focused on describing what made up good and bad SEIs from the viewpoint of health and care professionals and residents. Given the parity between staff and residents’ views, across time and place, it is hoped that the findings from this paper will be included within or used to shape SEI for health and care staff and support future mealtime improvement interventions. Future research could include the analysis of current supported eating training, examining if and how they are underpinned by research findings.

However, it will take more than the training of individual healthcare staff to make positive change in this area and consistently deliver positive SEIs. Despite the step change in staff knowledge and skills highlighted above, the ability to identify what makes up good SEIs and deliver them are two different things. Future research employing ethnographic, observational methods in these settings may be able to uncover whether both individual staff members and organizations can consistently put into practice the ‘good’ practice they identified in the interviews here.

This study’s findings demonstrate a continuous tension between medicalized practices and the clinical needs of residents versus the social nature of eating. Achieving a balance between both is described by staff and residents alike as being critical in achieving a good SEI. Achieving this in practice, however, seems to be challenging given the plethora of medicalized practices interrupting mealtimes. The idea of ‘protected mealtimes’, a practice often attempted in hospitals where visitors are restricted from entering the ward during mealtimes to keep the environment quieter could be applied to staff delivering medical interventions or interactions that can wait until after mealtimes in long term care. What prevents mealtimes for adults with neurological conditions residing in long-term care settings being ‘protected’ from medical interventions or clinical activity was not the aim of this paper but is an important question for future study.

Multiple barriers to the provision of a good SEI were highlighted by health and care professional participants. One such challenge was the threat to choice imposed by time pressures and funding outside of the long-term care facility. For those who return home or to other forms of community living, the time an SEI takes can be heavily bounded by the length of a short ‘care call’. Understanding more about and evidencing these restraints upon SEI’s and their impacts on cared-for individuals is an important area of future research and policy.

**Limitations of this study**

This study has drawn on and brought together both resident and health and care professional perspectives. Despite differences in the roles and positions of the interviewees, the giver of the food or the receiver, there was remarkable agreement across groups and care settings regarding what makes for good and bad SEI.

This study has uniquely provided a detailed and nuanced description of what makes up a good and bad supported eating interaction, yielding tangible actions for health and care professionals, or anyone who supports adults with neurological conditions to eat.

The study however draws upon the perspectives of residents and health and care professionals. It has not captured the experiences or observations of family members, who could provide an important additional viewpoint. The study utilises interview data only. It details the ideal SEI but does not evidence what happens in practice or how close or far away SEI’s are to these described ideals. Utilising ethnographic, observational methods to witness and describe SEI’s in action would expand understanding of what actually happens within SEI’s in day-day practice.

The study also focuses on the individuals within the interaction and has not examined the organisational, policy and political elements that shape and/or press upon these interactions.

**Clinical Implications**

Supported eating training should include considerations of time and timing, individualising the interaction, providing choice, how to maintain dignity, manage risk and the mealtime environment for any given resident.

Based on this study’s findings, organisations can improve SEI’s by a) reducing clinical activities, artefacts and discussions within the SEI and mealtime space, b) ensuring health and care staff are enabled to give residents the time they need when being supported to eat and c) reifying the SEI as a critical aspect of care provision within their organisation.

The findings from this paper could be considered for inclusion in care quality guidelines by care inspectorates who govern care quality in long-term care settings.

**Conclusion**

In conclusion residents and health and care professionals consider good SEI’s to be made up of well-paced interactions delivered by patient and caring health and care professionals who individualise the interaction, maintain dignity, provide residents with choice and have the clinical skills to manage the risks of choking or aspiration. Central to a safe and pleasurable SEI is its delivery in a well-managed mealtime environment where the right amount and type of stimuli has been carefully considered for those eating.

Patient practices of care required to deliver a positive interaction described within the findings here can be deemed as time intensive by care providers but defining it or viewing it as ‘task’ unhelpfully devalues its importance to residents being cared for. Reframing supported eating as a social activity and daily opportunity to demonstrate and provide high-quality care could begin a positive shift toward reifying mealtimes and recognizing and rewarding the skills needed to successfully deliver positive SEIs.

Research suggests that relatively small changes to mealtime delivery can have an impact on residents’ well-being and satisfaction (Barnes et al. 2013). Pursuing positive changes in this area is therefore an important and achievable target for improving the well-being of people with neurological conditions (and others) in long-term care facilities. Considering that one of the major sources of possible pleasure for people in these places is food, it is both a challenge and responsibility of health and social care providers to stimulate and maximize this enjoyment in residents for as long as possible.

**Funding**

This work has been supported by Health and Care Research Wales who funded study Food, Eating and drinking in neurological care: Sharing practice to Transform care (FEAST), Grant number SCF-18-1503, the study from which this paper draws.

**Declaration of interest statement**

The authors report there are no competing interests to declare.

**Biographical note**

Dr Julie Latchem-Hastings is a Senior Lecturer in the School of Healthcare Sciences at Cardiff University (https://profiles.cardiff.ac.uk/staff/latchemjm). A physiotherapist by background Julie's research focusses on the wellbeing, care and rehabilitation of people with long-term conditions - particular under researched communities/populations. She has a special interest in people with neurological conditions, particularly younger adults (18-65) with neurological conditions living in long-term care and people with a prolonged disorder of consciousness.

Dr Latchem-Hastings is Deputy Director of the Coma and Disorders of Consciousness Research Centre (www.cdoc.org.uk), a multi-disciplinary group of researchers based at Cardiff University exploring the cultural, ethical, legal and social dimensions of coma, vegetative and minimally conscious states. Dr Latchem-Hastings has carried out a range of studies in this area, including exploring how allied health professional treatments are perceived by families of people with a prolonged disorder of consciousness.

Dr Latchem-Hastings enjoys working with creative artists and using creative methods to engage with participants and the public in her research. For example, the findings from this paper have been made visual through a co-produced photography project and accompanying exhibition. She is interested in co-production and co-design and using her research to develop patient, public or allied health professional led interventions and resources.

**Data availability statement**

The data that support the findings of this study, where participants consented to data sharing for the purposes of secondary analysis, are available from the author upon reasonable request, and subject to a data sharing agreement between the authors and the requester institutions.

**Author contributions statement**

This paper is a single authored paper. The study from which this paper draw was part of a postdoctoral fellowship – a personal award. The author confirms sole responsibility for the study conception and design, data collection, analysis and interpretation of findings, and manuscript preparation.

**References**

Adams, K., Anderson, J., Archuleta, M., and Smith Kudin, J. (2013). Defining skilled nursing facility residents' dining style preferences. *Journal of Nutrition in Gerontology and Geriatrics*, 32(3), pp. 213–232.  DOI: [10.1080/21551197.2013.810560](https://doi.org/10.1080/21551197.2013.810560)

Athlin, E., Norberg, A., & Jansson, L. (1989). Feeding problems in severely demented persons seen from task and relationship aspect. *Scandinavian Journal of Caring Science*, 3(3), 113-121. DOI: [10.1111/j.1471-6712.1989.tb00383.x](https://doi.org/10.1111/j.1471-6712.1989.tb00383.x)

Athlin, E., Norberg, A., & Asplund, K. (1990). Caregivers’ perceptions and interpretations of severely demented persons during feeding in a task assignment system. *Scandinavian Journal of Caring Science*, 4(4), 147 155. DOI: [10.1111/j.1471-6712.1990.tb00065.x](https://doi.org/10.1111/j.1471-6712.1990.tb00065.x)

Backstrom, A., Norberg, A. and Norberg, B. (1987). Feeding difficulties in long-stay patients at nursing homes. Caregiver turnover and caregivers’ assessments of duration and difficulty of assisted feeding and amount of food received by the patient. *Int. Journal of Nursing Studies*. 24(1), pp. 69-76. DOI: [10.1016/0020-7489(87)90040-x](https://doi.org/10.1016/0020-7489(87)90040-x)

Barnes, S., Wasielewska, A., Raiswell, C. and Drummond, B. (2013). Exploring the mealtime experience in residential care settings for older people: an observational study. *Health and Social Care in the Community*, 21(4), pp. 442-450. DOI: [10.1111/hsc.12033](https://doi.org/10.1111/hsc.12033)

Braun, V and Clarke, V. (2022). *Thematic Analysis: A Practical Guide*. London: Sage.

Burgos, R., Bretón, I,, Cereda, E., Desport, J.C, Dziewas, R., Genton, L., et al. (2018). ESPEN guideline clinical nutrition in neurology. *Clin Nutr*. 37(1), pp. 354-396. doi: 10.1016/j.clnu.2017.09.003.

Top of Form

Bottom of Form

Carrier, N., West, G.E. and Ouellet, D. (2009). Dining experience, foodservices and staffing are associated with quality of life in elderly nursing home residents. *The Journal of Nutrition, Health & Aging*, 13 (6), pp. 565-570. DOI: [10.1007/s12603-009-0108-8](https://doi.org/10.1007/s12603-009-0108-8)

Chaudhury, H., Hung, L. and Badger, M. (2013). The role of physical environment in supporting person-centered dining in long-term care: A review of the literature American *Journal of Alzheimer's Disease and Other Dementias*, 28 (5), pp. 491-500. DOI: [10.1177/1533317513488923](https://doi.org/10.1177/1533317513488923)

Chou, H-H., Tsou, M-T and Hwag, L-C. (2020) Nasogastric tube feeding versus assisted hand feeding in home healthcare older adults with severe dementia in Taiwan: a prognosis comparison. *BMC Geriatric,* 20(1), pp.60. Doi: [10.1186/s12877-020-1464-9](https://doi.org/10.1186/s12877-020-1464-9)

Clarke, G., Fistein, E., Holland, A. *et al.* (2018). Planning for an uncertain future in progressive neurological disease: a qualitative study of patient and family decision-making with a focus on eating and drinking. *BMC Neurol* 18(115). https://doi.org/10.1186/s12883-018-1112-6

Davies, M., Zuniga, M., Verbeek, H and Staudacher (2022). Exploring resident experiences of person-centred care at mealtimes in long-term residential care: a rapid ethnography. BMC Geriatrics, 22(963).

Department of Health and the Nutrition Summit Stakeholder Group (2007). Improving Nutritional Care: A Joint Action Plan. Department of Health and Nutrition Summit Stakeholders, London, UK.

Feigin, V.L., Vos, T., Nichols, E., Owolabi, M.O., Carroll, W.M., Dichgans, M., Deuschl. G., Parmar, P., Brainin. M. and Murray C. (2020). The global burden of neurological disorders: translating evidence into policy. *Lancet Neurol.,* 19(3), pp. 255-265. DOI: 10.1016/S1474-4422(19)30411-9

GBD 2016 Neurology Collaborators (2019). Global, regional, and national burden of neurological disorders, 1990-2016: a systematic analysis for the Global Burden of Disease Study. *Lancet Neurol*., 18(5), pp. 459-480. doi: 10.1016/S1474-4422(18)30499-X.

Halfpenny, R., Stewart, A., Kelly, P. *et al.* (2021) Dysphagia rehabilitation following acquired brain injury, including cerebral palsy, across the lifespan: a scoping review protocol. *Syst Rev* **10**, 312 (2021). <https://doi.org/10.1186/s13643-021-01861-9>

Harstäde, C.W., Andersson, S., Lagerbielke, E. *et al.* (2025) Mealtime situations in nursing homes from the residents’ perspective – an integrative review. *BMC Geriatr* 25, 103. <https://doi.org/10.1186/s12877-025-05753-8>

Heaven, B., Bamford, C., May, C. and Moynihan, P. (2012). Food work and feeding assistance on hospital wards. *Sociology of Health and Illness,* 35(4), pp. 628-642 DOI: [10.1111/j.1467-9566.2012.01515.x](https://doi.org/10.1111/j.1467-9566.2012.01515.x)

Josefsson, M., Nydahl, M, Persson, I and Sydner, Y. (2018). Reforming foodservice in elderly care: National actions and local outcomes. *Nutrition & Dietetics,* 75(1), pp.79-86. DOI: [10.1111/1747-0080.12388](https://doi.org/10.1111/1747-0080.12388)

Kayser-Jones, J. and Schell, E. (1997). The effect of staffing on the quality of care at mealtime. *Nursing Outlook,* 45(2), pp. 64-72. DOI: [10.1016/s0029-6554(97)90081-6](https://doi.org/10.1016/s0029-6554(97)90081-6)

Kiddler, L.H and Fine, M. (1987). Qualitative and quantitative methods: When stories converge. In M.M Mark and L. Shotland (eds), New directions for program evaluation (pp. 57-77. San Francisco, CA: Jossey-Bass.

Kumlien, S., and Axelsson, K. (2002). Stroke persons in nursing homes: eating, feeding, nutrition and related care. *Journal of Clinical Nursing*, 11, pp. 498-509. DOI: [10.1046/j.1365-2702.2002.00636.x](https://doi.org/10.1046/j.1365-2702.2002.00636.x)

Latchem, J. (2017). *Shaping and sharing futures in brain injury rehabilitation*. P.h.D Thesis. Cardiff University. <https://orca.cardiff.ac.uk/108215/>

Latchem-Hastings, J. (2023). Poster Boys and the Rehabilitative Dream: Using a Temporal Lens to Explore Severe Brain Injury Rehabilitation. *Journal of Long-Term Care.* DOI: 10.31389/jltc.166

Lowndes, R., Armstrong, P. and Daly, T. (2015). The meaning of ‘dining’: The social organization of food in long-term care. *Food Studies*, 4 (1), pp. 19-34.

Manthorpe, J. and Watson, R. (2003). Poorly served? Eating with dementia. *Journal of Advanced Nursing,* 41 (2), pp. 162– 169.  DOI: [10.1046/j.1365-2648.2003.02520.x](https://doi.org/10.1046/j.1365-2648.2003.02520.x)

Martinsen, B., Paterson, B., Harder, I and Biering-Sørensen, F. (2007). The nature of feeding completely dependent persons: A meta-ethnography. *International Journal of Qualitative Studies on Health and Well-being*, 2:4, pp. 208-216, DOI:10.1080/17482620701296291

Martinsen, B., Harder, I. and Biering-Sorensen, F. (2008). The meaning of assisted feeding for people living with spinal cord injury: a phenomenological study. *Journal of Advanced Nursing* 62(5), pp. 533–540. doi: 10.1111/j.1365-2648.2008.04637.x

Merrell J., Philpin S., Warring J. and Hobby, D. (2012) Addressing the nutritional needs of older people in residential care homes. *Health and Social Care in the Community*, 20 (2), pp 208–215. DOI: [10.1111/j.1365-2524.2011.01033.x](https://doi.org/10.1111/j.1365-2524.2011.01033.x)

Michaelsson, E., Norberg, A., & Norberg, B. (1987). Feeding methods for demented persons in end stage of life. *Geriatric Nursing*, 8(2), pp. 69-73. DOI: [10.1016/s0197-4572(87)80091-5](https://doi.org/10.1016/s0197-4572(87)80091-5)

Morrison-Koechl, J., Wu, S.A., Slaughter, S.E., Lengyel, C.O., Carrier, N. and Keller. H. H. (2021). Hungry for More: Low Resident Social Engagement Is Indirectly Associated With Poor Energy Intake and Mealtime Experience in Long-Term Care Homes. *Appetite,* 159: 105044. DOI: [10.1016/j.appet.2020.105044](https://doi.org/10.1016/j.appet.2020.105044)

Namasivayam-MacDonald, A., Morrison, J., Steele, C., Keller, H. (2017). How Swallow Pressures and Dysphagia Affect Malnutrition and Mealtime Outcomes in Long-Term Care *Dysphagia*, 32(6), pp.785-796. DOI: [10.1007/s00455-017-9825-z](https://doi.org/10.1007/s00455-017-9825-z)

Nielsen, M., Werkander Harstäde, C. and Sandgren, A. (2025), Eating Together but Often Feeling Lonely: Residents' Mealtime Experiences in a Nursing Home. *J Clin Nurs*, 34, pp. 259-267. <https://doi.org/10.1111/jocn.17561>

Older People’s commissioner for Wales. (2014). A Place to call Home?: A review into the quality of life and care of older people living in care homes in Wales. Available at: https://olderpeople.wales/wp-content/uploads/2022/05/A-Place-to-Call-Home-A-Review-into-the-Quality-of-Life-and-Care-of-Older-People-living-in-Care-Homes-in-Wales.pdf

Panebianco, M., Marchese-Ragona, R., Masiero, S. and Restivo, D.A. (2020) Dysphagia in neurological diseases: a literature review. *Neurol Sci*. 41(11), pp. 3067-3073. doi: 10.1007/s10072-020-04495-2.

Pasman, H.R, The, B.A, Onwuteaka-Philipsen, B.D, van der Wal, G and Ribbe M,W. (2003). Feeding nursing home patients with severe dementia: a qualitative study. *J Adv Nurs*. May;42(3):304-11. DOI: 10.1046/j.1365-2648.2003.02620.x.

Pearson, A., Fitzgerald, M. and Nay, R. (2003). Mealtimes in nursing homes: The role of nursing staff. *Journal of Gerontological Nursing*, 29(6), pp. 40-47. DOI: [10.3928/0098-9134-20030601-09](https://doi.org/10.3928/0098-9134-20030601-09)

Pellitier, C. A. (2005). Feeding beliefs of certified nurse assistants in the nursing home. A factor influencing practice. Journal of Gerontological Nursing, 31(7), pp. 5-10. DOI: [10.3928/0098-9134-20050701-04](https://doi.org/10.3928/0098-9134-20050701-04)

Pierson, C. A. (1999). Ethnomethodologic analysis of accounts of feeding demented residents in long-term care*. Journal of Nursing Scholarship*, 31(2), pp.127-131. DOI: [10.1111/j.1547-5069.1999.tb00447.x](https://doi.org/10.1111/j.1547-5069.1999.tb00447.x)

Philpin, S., Merrell, J., Warring, J., Hobby, D. and Gregory, V. (2014). Memories, identity and homeliness: The social construction of mealtimes in residential care homes in South Wales. *Ageing Soc*. 34(5), pp. 753–89.

Reimer, H.D and Keller, H, H. (2009). Mealtimes in nursing homes: striving for person-centered care. *J Nutr Elder*. 28(4):327-47. DOI: 10.1080/01639360903417066.

Royal Society for Public Health (2009). Eating for Health in Care Homes – A Practical Nutrition Handbook. RSPH, London, UK

Simmons, S. and Schnelle, S. (2006). Feeding assistance needs of long-stay nursing home residents and staff to provide care. *J Am Geritriac Soc,* 54(6), pp 919-24. DOI: [10.1111/j.1532-5415.2006.00812.x](https://doi.org/10.1111/j.1532-5415.2006.00812.x)

van Ort, S. and Phillips, L. (1992). Feeding nursing home residents with Alzheimer’s Disease. *Geriatric Nursing*, 13, pp. 249-253. DOI: [10.1016/s0197-4572(05)80413-6](https://doi.org/10.1016/s0197-4572(05)80413-6)

Wäckerlin, S., Gemperli, A., Sigrist-Nix, D. and Arnet, U. (2020) Need and availability of assistive devices to compensate for impaired hand function of individuals with tetraplegia. *J Spinal Cord Med*. 43(1), pp. 77-87. doi: 10.1080/10790268.2018.1479054.

Watkins R, Goodwin VA, Abbott RA, Hall A, Tarrant M. (2017). Exploring residents' experiences of mealtimes in care homes: A qualitative interview study. BMC Geriatr. 11;17(1):141. DOI: 10.1186/s12877-017-0540-2.
